# Review of Processing Pathological Vectorcardiographic Records for the Detection of Heart Disease

**DOI:** 10.3389/fphys.2022.856590

**Published:** 2022-03-21

**Authors:** Jaroslav Vondrak, Marek Penhaker

**Affiliations:** Faculty of Electrical Engineering and Computer Science, VSB-Technical University of Ostrava, Ostrava, Czech Republic

**Keywords:** vectorcardiography, heart disease, VCG features, transformation methods, electrocardiography

## Abstract

Vectorcardiography (VCG) is another useful method that provides us with useful spatial information about the electrical activity of the heart. The use of vectorcardiography in clinical practice is not common nowadays, mainly due to the well-established 12-lead ECG system. However, VCG leads can be derived from standard 12-lead ECG systems using mathematical transformations. These derived or directly measured VCG records have proven to be a useful tool for diagnosing various heart diseases such as myocardial infarction, ventricular hypertrophy, myocardial scars, long QT syndrome, etc., where standard ECG does not achieve reliable accuracy within automated detection. With the development of computer technology in recent years, vectorcardiography is beginning to come to the forefront again. In this review we highlight the analysis of VCG records within the extraction of functional parameters for the detection of heart disease. We focus on methods of processing VCG functionalities and their use in given pathologies. Improving or combining current or developing new advanced signal processing methods can contribute to better and earlier detection of heart disease. We also focus on the most commonly used methods to derive a VCG from 12-lead ECG.

## 1 Introduction

Measuring the electrical activity of the heart using electrocardiography and vectorcardiography is well established, as these methods have been used for more than a hundred years Burch (1985), Burch and DePasquale (1990). The cells that create the myocardium are joined together by gap junctions, which have very low resistance to a normal healthy heart. As a result, activity in one cell easily spreads to neighboring cells. However, this muscle cannot be controlled by the will. Durrer et al. (1970) published a study which shows that activation wavefronts progress relatively evenly, from endocardium to epicardium and from apex to base. Electrical activation of the heart begins in the sinus node (SA), and it spreads along the atrial walls. Then depolarization reach the atrioventricular (AV) node. Propagation of AV junction is very slow and results in delays during activation, which is a desirable, because it allows completion of ventricular filling. Once the activation reaches the chambers, the excitement continues along the Purkinje fibers. Furthermore, depolarization waves occur from left to right of the septum. Then, the depolarization spread through the left and right ventricular wall. Because the left ventricular wall is thicker, depolarization of the left ventricle continues even after depolarization of a large portion of the right ventricle. The left ventricle is depolarized mainly in parallel through the left anterior and posterior fascicles and the left the lateral basal part is the last to be activated. Ventricular repolarization begins on the outside of the ventricles and “spreads” inward. Although the epicardium is depolarized last, its action potential duration is short and it is the first to recover. Although single cell recovery does not propagate to neighboring cells, it can be noted that recovery generally moves from the epicardium to the endocardium. Inward repolarization generates a signal with the same sign as outward depolarization. Due to the diffuse form of repolarization, the amplitude of the signal is much smaller than the amplitude of the depolarization wave and lasts longer Malmivuo and Plonsey (1995). These changes in depolarization and repolarization are then measured from the patient’s body surface, most often in the form of a 12-lead ECG.

The method of vectorcardiography dates back to 1887, when in the first article concerning the human electrocardiogram, Augustus D. Waller pointed out the dipolar nature of the cardiac electric generator. Thus, it is possible to describe an electric generator of the heart with reasonable accuracy by an equivalent dipole. This dipole can be described as an electric heart vector (EHV) and it is possible to display it in vector form Waller (1887). In 1920s Mann first introduced the concept of a “loop” representing a continuous series of vectors for depicting electrical depolarization and repolarization magnitudes. Mann derived these loops manually from three Einthoven leads Burch (1985). From 1936 to 1940 the technique of vector representation of the electric field of the heart was actively developed in Germany Howard (1946). For direct measurement of orthogonal leads, Schellong et al. (1937) introduced the first uncorrected orthogonal system in 1937. Their work was further developed by other authors who defined new lead systems Kimura (1939), Duchosal and Sulzer (1949), Grishman et al. (1951), Milnor et al. (1953). These lead systems differ by placing the electrodes on the hull and are represented by signals that are orthogonal to each other. However, they do not take into account the different torso geometry or intrinsic tissue inhomogeneity. The first corrected lead system was derived by Frank based on a mathematical model Frank (1956). This system, which uses seven measuring electrodes, is today one of the most widely used vectorcardiographic leads. Other published but less commonly used vectorcardiographic leads include McFee and Parungao (1961), SVEC III Schmitt and Simonson (1955), and hybrid lead systems Dellborg et al. (1995).

**FIGURE 1:**
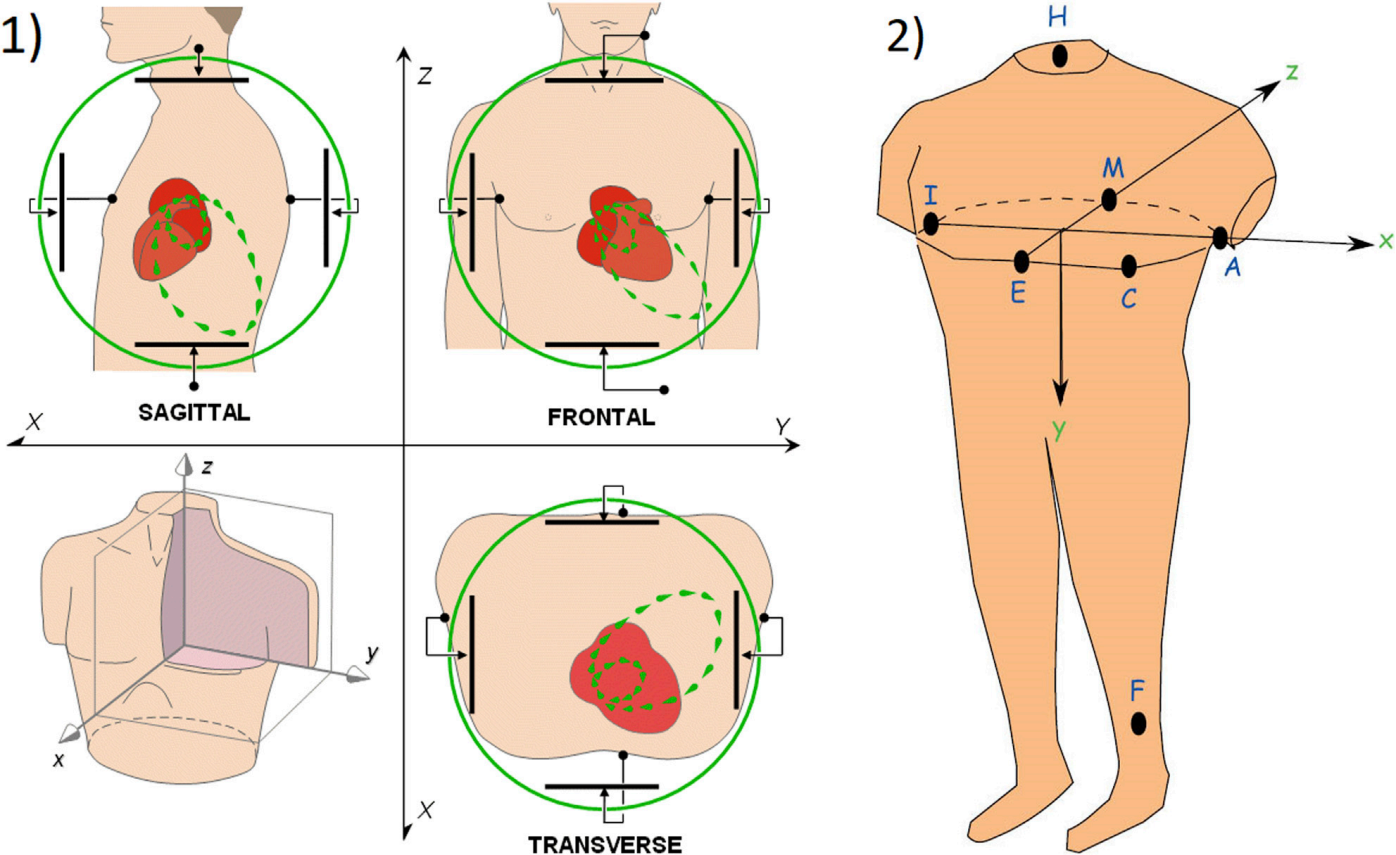
1) The basic principle of vectorcardiography is illustrated on ideal uniform lead fields, which are perpendicular to each other and are in a bipolar configuration (set by parallel electrodes on opposite sides of the torso) Malmivuo and Plonsey (1995); 2) Placement of measuring electrodes on the patient body using Frank lead system Hasan and Abbott (2016).

**FIGURE 2:**
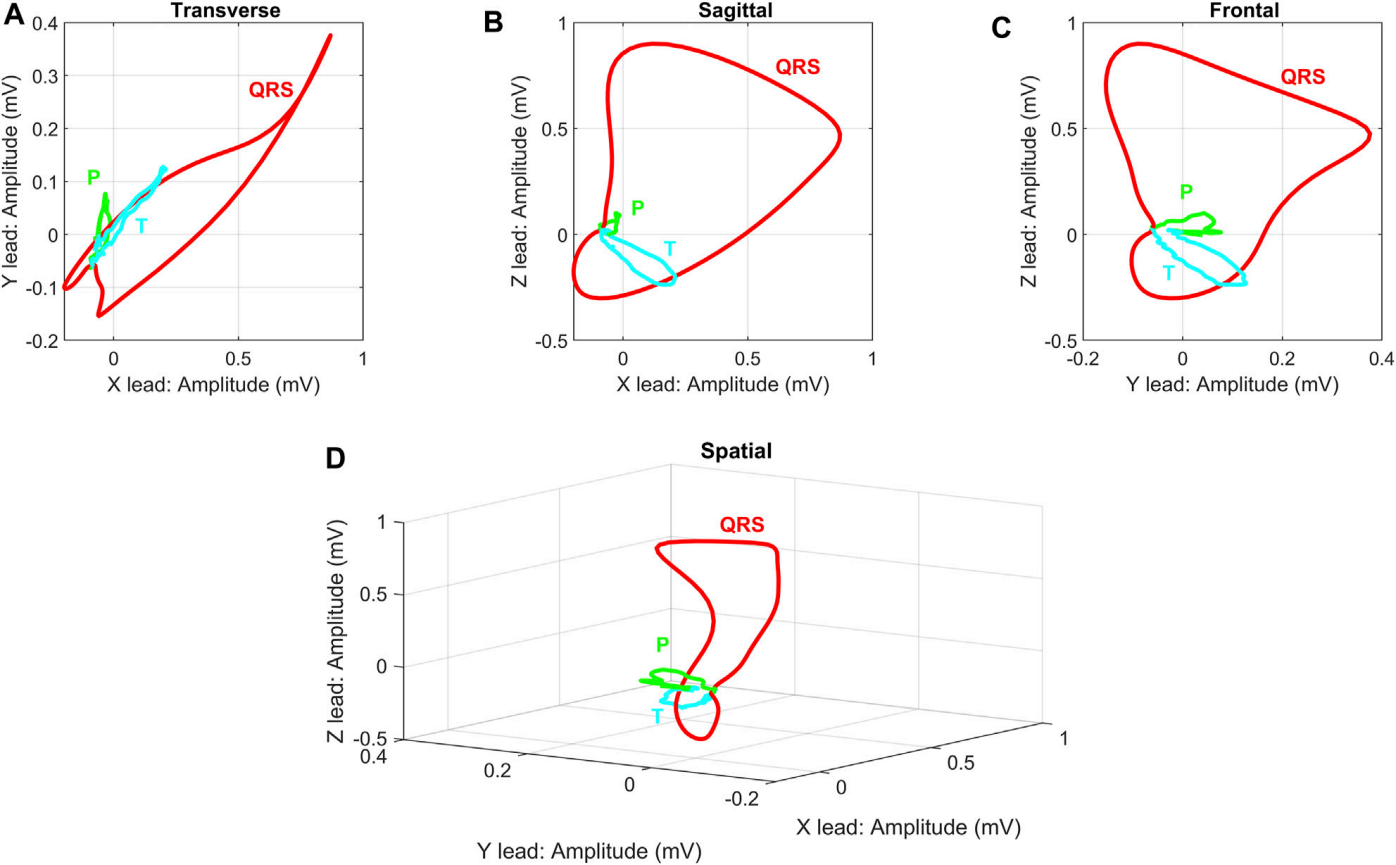
Demonstration display of individual VCG planes for randomly selected physiological record: **(A)** Transverse plane of X and Y leads, **(B)** Sagittal plane of X and Z leads, **(C)** Frontal plane of Y and Z leads, **(D)** 3-D image of X, Y and Z leads. The record *s*0503_*rem*_ from the PhysioNet PTB database was used as a randomly selected physiological record. The individual planes are related to the basic principle of VCG measurement on ideal uniform lead fields from Figure 2.

Like the ECG, VCG is a diagnostic method which is considered as a very useful method for measuring the electrical activity of the heart. It is more sensitive than a standard 12-lead ECG and provides the cardiologist with important additional information such as a clearer indication of the phase relationships between leads Rubel et al. (1991), Levkov (1987). Today, the QRS-T angle is the most commonly analyzed of the VCG, while current ECG markers of repolarization abnormalities mainly include ST depression, T wave inversion, and QT prolongation Dilaveris et al. (2001), Voulgari and Tentolouris (2009). The VCG is projected into three mutually perpendicular planes: sagittal, transversal and frontal, see Figure 1 (left), where placement of the electrodes are shown in Figure 1 (right). The individual planes are most often located as: frontal plane between X and Y leads, transversal plane between X and Z leads and sagittal plane between Y and Z leads. Cardiac activity is then described by three loops, which represent the individual phases of the cardiac cycle. The first loop corresponds to wave P, the second loop which is the largest corresponds to QRS complex and the third loop corresponds to wave T. The loops can be seen in three 2-D projections or in one 3-D image in a demonstrative physiological record from the PTB database, see Figure 2. The records contained in this database are sampled by sampling frequency of 1 kHz with a 16-bit resolution in the range of ±16,384 mV. Based on the recommendation Merri et al. (1990), the sampling frequency for measuring electrical activity of the heart should be at least 128 Hz. However, setting parameters in the PTB database is suitable for subsequent and detailed analysis of the electrical activity of the heart. However, along with the required signal, interfering components that need to be removed from the records can also be measured. When designing filters, it is necessary to take into account the frequency range of the desired signal and the frequency characteristics of individual filters.

The benefits of a 12-lead ECG for diagnostic evaluation of electrical activity of the heart has been a standard in clinical practice over a hundred of years. However, there are certain cases where the vectorcardiogram is superior to the electrocardiogram. In some publications, a higher sensitivity of VCG compared to conventional ECG has been reported in the diagnosis of atrial enlargement and right ventricular hypertrophy. It has been proposed to re-evaluate the frequency of 12-lead ECG usage to increase vectorcardiography measurements in clinical practice van Bemmel et al. (1992), Chou (1986). In recent years, VCG has become a method that is processed by modern signal processing procedures, mainly due to the possibility of obtaining and subsequent analysis of spatial features. Studies have shown that the vectorcardiogram is very useful in some specific situations, such as assessing intraventricular conduction disorders combined with inactive areas, identification of sudden cardiac death, identifying and locating ventricular preexcitation, differential diagnosis of patterns different from normal deviation from electrical axis, assessment particular aspects of Bruges’ syndrome and estimating the severity of some cardiac enlargements Sur et al. (2013), Perez Riera et al. (2007). Also, more accurate results were obtained in the analysis of QRS in three-dimensional projection, such as improved patient selection for cardiac resynchronization therapy (CRT), detection of myocardial injury Correa et al. (2010) or extraction of VCG features from QRS complex Correa et al. (2012) for ischemia detection. Another advantage of vectorcardiography over standard ECG is in detection of long QT syndrome Diamant et al. (2013, 2010), Cortez et al. (2017a). Another use of VCG, due to its higher sensitivity, was in the analysis of inducted cardiac memory, which was mainly known from ECG recordings. Wecke et al. (2013) analyzed the VCG records of patients after previous ablation of accessory pathways (AP). They were aware of the phenomenon of induced cardiac memory, which was present on ECG records in different patient ratios. They assumed that VCG, which is more sensitive than ECG, would show cardiac memory independent of AP location after ablation. They found out from the analyzed records that after ablation there was a correlation between the directions of the overexcited maximum QRS vector and the post-ablation maximum T-vector, indicating the presence of cardiac memory. From their findings, it was confirmed that the information we know from the ECG can also be found in the VCG with the possibility of a new perspective.

Vectorcardiography, which represents a slightly different approach, is used less commonly in practice and VCG leads are often derived Frank (1956), Iwaniec et al. (2018), Sedaghat et al. (2016), Sun et al. (2017), Treskes et al. (2015), Correa et al. (2012, 2013). This examination method can be considered as a useful tool in the study of many heart diseases and can provide additional information to the conventional ECG in the form of additional spatial information Vozda and Cerny (2015). However, VCG is not usually recorded in clinical practice but orthogonal leads can be derived from a conventional 12-lead ECG Belloch et al. (2007). The importance of vectorcardiography has been published in numerous publications, but VCG records are not available in most cases. Therefore, an alternative form of deriving VCG from a commonly measured 12-lead ECG was proposed. Derived VCG is useful for estimating some meaningful features that represent high diagnostic information such as QRS-T angle or Total cosine R to T (TCRT). These and other features can be estimated from derived VCG with sufficient accuracy Karsikas et al. (2009), Cortez and Schlegel (2010), Cortez et al. (2014).

In recent years, vectorcardiographic recordings have been increasingly used for the analysis and detection of heart disease. If directly measured records using Frank lead system are not used, they are often transformed by various methods from a 12-lead ECG. In the following chapter, this paper provides an overview of the most commonly used transformation methods. In addition, it reviews the processing and extraction of important diagnostic parameters, the most promising techniques and current challenges for the detection of various heart diseases.

## 2 Review Strategy

This work presents a comprehensive overview of the multidisciplinary area of vectorcardiographic record processing and methods of possible transformation from 12-lead ECG to obtain derived VCG leads. The individual methods and steps for the implementation of this overview are presented here. These are mainly: selection of suitable databases, selection of search terms, and evaluation of results. This review was conducted using full papers, including publications in journals, conference papers, books, and academic papers. The search was performed without a time limit to provide a historical background. The search for relevant works was carried out in English.

### 2.1 Database Selection

Four basic databases were selected for the classification of suitable literature. These include the Scopus database and the Web of Science, which are among the largest databases, including peer-reviewed citation sources. PubMed and ProQuest databases, which focus on the selection of medical and medical literature, were also used. The combination of medical and technical literature should be the basis for comprehensive information, both medical and especially technical in the field of vectorcardiographic processing.

### 2.2 Indexed Terms

In this section, we list the individual indexed terms that have been used for this overview. In the case of processing vectorcardiographic records, the terminology is inconsistent. Therefore, several combinations of indexed terms have been used to include the widest possible range of articles that are relevant to this review. A summary of the individual indexed terms used can be seen in Table 1.

**TABLE 1:** Indexed terms and their combinations.

Index terms	
1.	Vectorcardiography OR Vector Cardiography OR Vector Electrocardiography
2.	VCG OR ECG
3.	Transformation methods OR Derivation methods OR Linear transformation methods OR Quasi-orthogonal transformation OR Frank lead system
4.	VCG features OR ECG features
5.	P-loop OR QRS-loop OR T-loop
6.	Medical signal processing OR Biomedical signal processing

## 3 Transformation Methods

The first consideration on the transformation of individual lead systems was presented by Burger et al. (1952). Kornreich et al. (1974) described that the 12-lead ECG and Frank lead system were very similar in terms of their information content and therefore their mutual transformation was possible. This resulted in the first attempts to transform lead systems based on the transformation from VCG to 12-lead ECGs by Dower Dower (1968). Wolf et al. (1976) pointed out that knowledge from measuring of orthogonal lead systems can contribute to better diagnosis. He therefore considered the possibility of a two-way transformation and later he derived transformation matrices for the bidirectional transformation of conventional 12-lead ECG to VCG.

Many of the transformation methods were derived only for the QRS complex of the heart cycle. There are also transformation matrices that are focused on other parts of the heart cycle. For example, Guillem et al. (2006) designed a modified transformation matrix optimized for the P wave, thereby achieving improved transformation for this ECG segment. Guillem et al. (2008) addressed the issue of accuracy of P wave derivation further in. The object of this study was to test the accuracy of the inverse Dower transformation in comparison with the P-wave optimized transform. The problem of P-wave accuracy in a transformed VCG was also addressed by Carlson et al. (2005). In their study, they used a total of 41 patient records, of which 20 records were diagnosed with atrial fibrillation. After the transformation using the inverse Dower transformation, the waveform of P was preserved. When comparing the directly measured VCG and the derived VCG, the morphological parameters of the P wave were consistent in the respective groups, and better conservation was observed in the healthy groups.

Transformation methods for the derivation of ECG and VCG leads are an integral part of obtaining further beneficial information from the measurement of electrical activity of the heart. Most of the published articles use databases with already measured ECG and VCG records. However, simultaneously measured orthogonal data with a 12-lead ECG is not always available. Therefore, this chapter is devoted to the most commonly used transformation methods used in publications and scientific works.

### 3.1 Quasi—Orthogonal Transformation

Any ECG lead system can be converted into vectorcardiographic loops. However, these derived VCG loops will not be orthogonal. Certain leads from a 12-lead ECG show a high correlation with orthogonal leads. These leads are called as quasi-orthogonal. Such derived lead systems correspond approximately to uncorrected VCG leads. Correction can be achieved using geometry based on the torso model derived by Frank (1954).

Bjerle and Arvedson (1986) derived some of the first quasi-orthogonal leads that can be expressed as (Eq. 1):

$$\begin{array}{cc} & X=1,06\cdot V6\\ & Y=1,88\cdot VF=1,25\cdot aVF\\ & Z=-0,532\cdot V2+0,043\cdot V6 \end{array}$$ (1)

Kors et al. (1986) analyzed ECG lead systems and selected those leads that showed the highest correlation with orthogonal leads. Derived orthogonal leads can be expressed as Eq. 2:

$$\begin{array}{cc} & X=V6\\ & Y=II\\ & Z=-0,5\cdot V2 \end{array}$$ (2)

Kors et al. (1990) compared two quasi-orthogonal lead systems (Eq. 1) and (Eq. 2) and VCG derived by regression and Inverse Dower Transformation (IDT). Based on the measurement of the mean absolute deviation and the evaluation of cardiologists, he concluded that the transformation matrix obtained by regression and IDT achieved better results than both quasi orthogonal leads.

### 3.2 Transformation Methods Based on Linear Approach

The standard 12-lead ECG does not provide much information about the sagittal plane, so it is necessary to use all the information contained in the ECG to derive the VCG. It is the VCG that provides us information of cardiac activity in all three planes. The simplest conversion to a VCG is to use quasi-orthogonal conversions, where one ECG lead corresponds to one VCG lead. A more reliable variant is the use of linear transformation methods, where each ECG lead (I, II, V1, V2, … , V6) contributes to a certain extent to a specific orthogonal lead. The individual weight coefficients then form the resulting transformation matrix M. This approach was first introduced by Burger et al. (1952) in the analysis of the description of the electrical action of the heart by one dipole. Transformation matrix coefficients were derived using a regression approach and their accuracy tested in 169 patients. However, the authors did not publish the transformation coefficients.

For the conversion, the IDT has become one of the most widely used transformations. The coefficients can therefore be applied to individual ECG leads in the form of (Eq. 3).

$$\begin{array}{cc} & X=0,156\cdot I-0,01\cdot II-0,172\cdot V1-0,074\cdot V2\\ & +0,122\cdot V3+0,231\cdot V4+0,239\cdot V5+0,194\cdot V6 \end{array}$$ (3)

The Y and Z leads can be derived similarly. The difference between a quasi-orthogonal systems and a linear transformation can be noticed here. Individual VCG leads in a quasi-orthogonal system correspond to exactly one ECG lead, while in the case of a linear transformation matrix the VCG leads is formed by the weight coefficients of the individual ECG leads. The mathematical transformation is then realized as a multiplication of two matrices (Eq. 4).

V=M⋅E (4)

where M is the transformation matrix, E is matrix whose rows are formed by independent ECG leads and V is matrix whose rows correspond to the derived VCG.

The coefficients of the transformation matrices are also derived on the basis of the torso model described by Frank (1954) or by regression methods based on data measured in a representative group of patients. These coefficients then differ from one approach to another, describing in particular the morphology of the average patient or torso model.

### 3.3 Kors Regression Transformation

The transformation matrix introduced by Kors et al. (1990) was derived by regression technique for a group of patients from the CSE database. The transformation matrix coefficients, see Table 2, were derived by minimizing the mean error between the measured VCG and the transformed VCG.

**TABLE 2:** Transformation coefficients of Kors regression method.

Lead	I	II	V1	V2	V3	V4	V5	V6
X	0.38	−0.07	−0.13	0.05	−0.01	0.14	0.06	0.54
Y	−0.07	0.93	0.06	−0.02	−0.05	0.06	−0.17	0.13
Z	0.11	−0.23	−0.43	−0.06	−0.14	−0.20	−0.11	0.31

In this way, Kors derived several transformation matrices for different complex segments. He also stated that the differences between individual transformation matrices are small. Given that the QRS complex is most often analyzed, the resulting matrix is based only on the regression of the QRS complex.

Several publications have studied the transformation method introduced by Kors. The authors in Cortez and Schlegel (2010) and Cortez et al. (2014) discussed which of the available transformation methods provides the QRS-T spatial angle value closest to the values obtained from Frank lead system. They used two available transformation matrices: the Kors regression transform and the IDT. The authors concluded that the resulting values from the Kors regression method did not differ significantly from the values from Frank lead system. In their further publications, the authors in Cortez et al. (2015) have focused on the analysis of the spatial angle of QRS-T in patients with hypertrophic cardiomyopathy. Similarly, Man et al. (2011) analyzed the QRS-T spatial angle from the derived VCG data using the Kors regression method and IDT. Of the two transformation methods used, the Kors regression method achieved better results. The analysis was performed between the transformed VCGs and the VCGs measured by the Frank lead system.

### 3.4 Inverse Dower Transformation (IDT)

In 1980, Dower et al. (1980) presented the possibility of deriving 12-lead ECGs from three leads measured by the Frank lead system. They created the transformation coefficients, see Table 3, which provide better correlation for precordial electrodes with respect to the voltage and P and T waves in leads V1 and V2 Dower (1968); Dower et al. (1980); Dower and Machado (1979).

**TABLE 3:** Leading vectors for deriving a 12-lead electrocardiogram from the Frank XYZ signal.

Lead	I	II	III	aVR	aVL	aVF	V1	V2	V3	V4	V5	V6
X	0.632	0.235	−0.397	−0.434	0.515	−0.081	−0.515	0.044	0.882	1.213	2.125	0.831
Y	−0.235	1.066	1.301	−0.415	−0.768	1.184	0.157	0.164	0.098	0.127	0.127	0.076
Z	0.059	−0.132	−0.191	0.037	0.125	−0.162	−0.917	−1.387	−1.277	−0.604	−0.086	0.230

Using this knowledge, Edenbrandt and Pahlm (1988) derived a pseudo-inverse matrix that can be used for transformation from 8 independent ECG leads to VCG. The resulting pseudoinverse matrix is shown in Table 4.

**TABLE 4:** Transformation matrix for Inverse Dower transformation (IDT).

Lead	I	II	V1	V2	V3	V4	V5	V6
X	0.156	−0.010	−0.172	−0.074	0.122	0.231	0.239	0.194
Y	−0.227	0.887	0.057	−0.019	−0.106	−0.022	0.041	0.048
Z	0.022	0.102	−0.229	−0.310	−0.246	−0.063	0.055	0.108

The Inverse Dower Transformation was used by Panagiotou et al. (2013) and Dima et al. (2013) to transform a 12-lead ECG into a VCG for subsequent feature analysis to detect myocardial scar. Similarly, Sun et al. (2017) used IDT to analyze the VCG loop in patients with myocardial ischemia. Dawson et al. (2009) compared the Dower transformation matrix with the affinity transform in relation to the transformation from 12 to 8 lead ECG to 3 lead VCG and back. Based on the evaluated results, they conclude that in both myocardial infarction (MI) and healthy (HC) patients, the affinity transformation achieves better results in transformation from 3-lead VCG to 12-lead ECG than the Dower transform.

### 3.5 P Least Square Value (PLSV) and Q Least Square Value (QLSV) Transformations

Transformation matrices derived from the regression approach mainly focus only on the QRS complex of the heart cycle. The accuracy of P and T waves is usually considered sufficient in transformations and the differences in transformation matrices are minimal. VCG loops can be used to detect arrhythmias with high accuracy, emphasis is also placed on the accuracy of P and T wave transformation Kors et al. (1990).

Guillem et al. (2006) presented a transformation matrix derived using the regression method, which is optimized for P wave. They named the transformation matrix as PLSV, see Table 5. In addition to the matrix targeting the P wave, they also derived a matrix optimized only for the QRS complex, transformation coefficients are shown in Table 6. Both of these matrices were derived from a total of 124 patients. Using the least squares method, a regression model was found for each patient and the resulting matrix is given as the mean value of the transformation matrices for all patients. Guillem et al. (2006) also compared the Kors and PLSV matrices for atrial fibrillation records, and the PLSV transform yielded significantly better results in this regard.

**TABLE 5:** PLSV transformation matrix.

Lead	I	II	V1	V2	V3	V4	V5	V6
X	0.370	−0.154	−0.266	0.027	0.065	0.131	0.203	0.220
Y	−0.131	0.717	0.088	−0.088	0.003	0.042	0.048	0.067
Z	0.184	−0.114	−0.319	−0.198	−0.167	−0.099	−0.009	0.060

**TABLE 6:** QLSV transformation matrix.

Lead	I	II	V1	V2	V3	V4	V5	V6
X	0.199	−0.018	−0.147	−0.058	0.037	0.139	0.232	0.226
Y	−0.164	0.503	0.023	−0.085	−0.003	0.033	0.060	0.104
Z	0.085	−0.130	−0.184	−0.163	−0.193	−0.119	−0.023	0.043

### 3.6 Transformation From Mason-Likar (ML) ECG Leads

During stress tests, measuring a 12-lead ECG is not appropriate due to limb movement. Therefore, Mason and Likar have published their recommendations on how to limit the movement of electrodes when measuring ECG in stress tests Mason and Likar (1966). Interfering components that are created by movement are eliminated by moving the measuring electrodes to the chest. The resulting differences in signals should always be taken into account Papouchado et al. (1987). For these reasons, standard linear transformation methods cannot be used. Guldenring et al. (2012) designed a new transformation matrix, see Table 7, for the transformation of ECG measured using Mason-Likar leads.

**TABLE 7:** Mason–Likar transformation matrix.

Lead	I	II	V1	V2	V3	V4	V5	V6
X	0.5169	−0.0722	−0.0753	0.0162	0.0384	0.0545	0.1384	0.4606
Y	−0.2406	0.6344	0.1707	−0.0833	0.1182	0.0237	−0.1649	0.2100
Z	−0.0715	−0.1962	−0.4987	−0.0319	−0.2362	−0.0507	−0.2007	0.4122

### 3.7 Singular Value Decomposition of 12-Lead ECG

Another possibility of deriving vector cardiographic leads is by reducing the dimension of data using Singular Value Decomposition (SVD). This is an orthogonal matrix reduction of the data dimension defined by Golub and Van Loan (1996). The principle of this transformation is as follows (Eq. 5):

$$\Sigma =U^{T}MV$$ (5)

where columns of U are referred to as the left singular vectors, columns of V are referred to as the right singular vectors and M is 8 x *n* matrix of individual ECG lead of *n* samples Acar et al. (1999).

The orthogonal leads obtained in this way do not correspond directly to the Frank VCG, and the indications obtained from these leads do not correspond to the indications derived from the VCG leads, as stated by Belloch et al. (2007). However, this method found use Acar and Koymen (1999) where Hasan et al. (2012a,b) analyzed the morphology of QRS-T loops using SVD.

### 3.8 Summary of Transformation Methods

Transformation methods were derived to obtain orthogonal lead leads from a 12-lead ECG. There are many linear transformation methods that are used in various branches of VCG processing. However, each method has its advantages and disadvantages, especially in the processing of pathological records, where different pathologies affect different parts of the heart cycle. In such a case, knowledge of the effect of pathology on the ECG would be required to select the correct transformation method. If a transformation method is selected that is not suitable for a particular part of the ECG recordings, diagnostic information may be lost by signal distortion. The following Table 8 summarizes the key features of each linear transformation method. The Accuracy column shows the accuracy of the transformation method according to the evaluation parameter of correlation in individual leads. This is one of the most frequently used parameters in the evaluation of transformation methods. The stated quantitative values are defined as average values of all leads from publications Jaros et al. (2019), Vozda and Cerny (2015), Kors et al. (1990). However, these values are only indicative, as the accuracy of the derivation depends on several factors.

**TABLE 8:** Overview of transformation methods.

Transformation method	Derivation of transformation methods	Primary use	Accuracy
Kors regression transformation	Minimizing the mean error between the measured VCG and the transformed VCG	All types of ECG	> 98%
Inverse Dower transformation (IDT)	Pseudo-inverse matrix to a system based on a torso model	Pathology affecting the QRS section	> 97.2%
PLSV transformation	Derivation by least squares method	P wave of ECG	> 96.8%
QLSV transformation	Derivation by least squares method	QRS complex of ECG	> 97%
Quasi-orthogonal transformation	Approximation to VCG leads from ECG leads	All types of ECG	> 90%
Mason-Likar (ML)	Designed using the regression method	Exercise and movement ECG	> 95%

One of the most frequently used transformation method is the IDT followed by the Kors regression method. There are more and more studies that point to the fact that Kors regression method achieves higher accuracy than other transformations Cortez and Schlegel (2010), Cortez et al. (2014), Man et al. (2011), Kors et al. (1990). The QLSV and PLSV methods are mainly focused on a certain part of the ECG (QLSV—QRS complex, PLSV—P wave). The quasi orthogonal method is derived by approximation to VCG leads and is not suitable for processing due to its high error rate.

**FIGURE 3:**
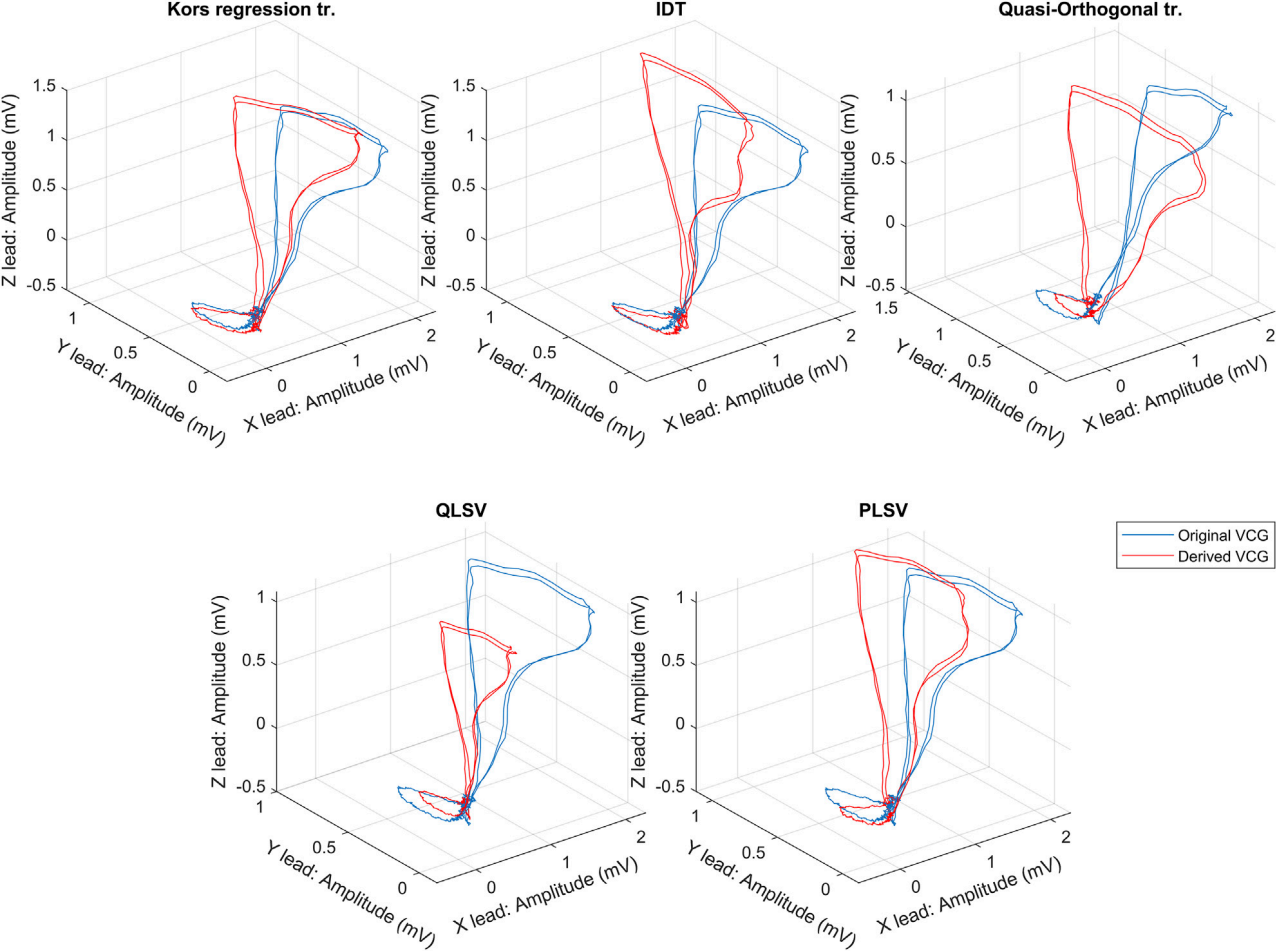
An exemplary comparison of transformation methods, where the blue curve is measured by the Frank lead system and the red curves are the transformed one.

If we assume a pathology that will affect more parts of the ECG, a combination of two or more transformation methods could be used to obtain a more accurately derived VCG. A graphic comparison of the individual transformation methods can be seen in Figure 3. From the first point of view, it can be seen that the Kors regression method copies the shape of the directly measured curve most accurately. However, various statistical tests are needed to verify the accuracy of the individual methods. Nevertheless, in the case of greater use of Frank’s lead system in clinical practice to obtain the original VCG, it would not be necessary to address the possibilities of transformation and their accuracy in the preservation of diagnostic information.

The following chapter describes the possibilities of detecting individual heart diseases from VCG records. Because VCG is not commonly obtained in clinical practice, transformation methods are being approached. These transformation methods are also listed in the individual publications when used.

## 4 Heart Disease Analysis From Vectorcardiography

Since the 19th century, it has been known that the heart muscle emits quasi-periodic electrical signals during its activity. Although it is very important, there are no standardized methods for detecting pathologies from electrical heart activity measured in three mutually perpendicular planes. This is mainly due to the poor use of VCG in clinical practice and the scientific work that does not provide the necessary evaluation from cardiologists. Greater cooperation between authors and relevant doctors and mutual openness to new procedures could address this shortcoming.

Using various methods, it is possible to find a pattern of healthy cardiac rhythms and different patterns for each type of hearth disease. Published contributions and studies evaluate patient records using various methods. It is important to use the right criteria for an objective comparison of the new methods. This section presents the different methods and techniques used for the subsequent evaluation of the most frequently analyzed pathologies.

### 4.1 Acute Cardiac Ischemia

Acute cardiac ischemia can be characterized as an imbalance between myocardial oxygen supply and demand. The reduction in blood circulation is progressive and by delivering insufficient blood to the myocardium are myocardial cells deprived of the blood combinations necessary for their survival Tomey and Gidwani (2016). The authors approached to the detection of these life-threatening conditions by various methods of processing VCG records, where the most common cases were mainly myocardial ischemia and myocardial infarction.

#### 4.1.1 Myocardial Ischemia

In recent years, several publications have promoted different methods for detecting and classifying electrical cardiac activity in patients with myocardial ischemia Persson et al. (2006), Martínez et al. (2006), Arini et al. (2008), Toledo and Wagner (2007). Several publications have dealt with the topic of preventing these life-threatening conditions by early detection, where authors approach different methods of VCG analysis. The detection of myocardial ischemia was dealt by Sun et al. (2017), where they focused on the development of a screening system using a vectorcardiogram. They used a total of 132 patient ECG records from two databases and introduced a total of 14 new characteristics to detect arrhythmia. They used derived VCGs using IDT to analyze VCG features. Accuracy of the proposed method was verified by calculating Accuracy (Acc) (Eq. 6), Sensitivity (Sens) (Eq. 7) and Specificity (Spec) (Eq. 8). The same calculation procedure was also used by Dima et al. (2013), Sun et al. (2017), Correa et al. (2013), Yang et al. (2012), Correa et al. (2016), Yang et al. (2013), Tripathy and Dandapat (2017), Huang et al. (2011).

$$Sens=\frac{TP}{TP+FN}$$ (6)

$$Spec=\frac{TN}{TN+FP}$$ (7)

$$Acc=\frac{TP+TN}{FN+FP+TP+TN}$$ (8)

where True Positive (TP)/False Negative (FN) are records that have a Ischemic Heart Disease (IHD) and are correctly/incorrectly identified, while False Positive (FP)/True Negative (TN) are records that do not have a IHD and are incorrectly/correctly classified. The accuracy of the proposed method reached 98.07% sensitivity 98.63% and specificity 99.04%. They state that the reliability of the proposed method is guaranteed by the analysis of pathophysiology and application of QRS, ST and T criteria. Another processing of QRS area connected with ST segment was presented by Kawahito et al. (2003). They analyzed the difference in the QRS vector, which reflects changes in the shape of the QRS complex, and the size of the ST vector, which represents the diversion of the ST segment from the isoelectric level. They states that monitoring the ST vector size and the QRS vector difference by vectorcardiography may be useful for identifying myocardial ischemia during carotid endarterectomy.

Correa et al. (2012) analyzed vectorcardiographic curves for the detection of ischemia and used a total of 80 ischemic and 52 healthy records. They studied five parameters, where the best results were achieved with a QRS Volume with a sensitivity of 64.5%, a specificity of 74.6% and an Area Under Receiver Operating Characteristic Curve (AUC) = 0.77. Later, they expanded their work, where their objective was evaluating the vectorcardiographic difference between both groups Correa et al. (2013). Synthesized orthogonal leads were obtained by Kors transformation. They analyzed seven QRS loop parameters, and from their analysis the best results achieves a QRS volume, which achieved sensitivity 64.5% and specificity 74.6%. In conclusion, they emphasize the fact that VCG and ECG parameters have significant differences between healthy and ischemic subjects. The QRS analysis was extended by Sun et al. (2017) by evaluation of ST and T segment. They analyzed a total of 17 parameters from the modified VCG using the IDT. Out of the tested number of 132 records they achieved an accuracy of 98.07%, a sensitivity of 98.63% and a specificity of 99.04%.

Dehnavi et al. (2011) used the Principal Component Analysis (PCA) and Independent Component Analysis (ICA) methods to reduce extracted VCG features. They used a set of 60 ischemic and 10 physiological records and extracted a total of 22 VCG parameters. After the reduction, they acquired five features that served as parameters for input to the neural network classifier (NNC). Their designed model has achieved accuracy 86% in distinguishing HC from MI. They also used the classical method of IHD detection from ECG parameters (ST segment and T-wave morphology) and compared to VCG parameters, detection from ECG achieves lower accuracy (73%). Based on the results obtained in this study authors indicate that VCG has higher accuracy and sensitivity in automated detection IHD than ECG.

Schuepbach et al. (2008) used cardiogoniometric leads (*VCG*_*C*_) and classifies healthy (HC) and ischemic (IHD) patients. Coronary angiography (CA) was used as a reference method. They describe VCG as a set of parameters including: time scale, size and direction of vectors, ratios of R/T vectors and ST/T segments, and the percentage location of QRS and T loops in space. A follow-up article by Seeck et al. (2009) already uses Linear Discriminant Functional Analysis (LDA) and the Machine Learning Method for classification. Huebner et al. (2010) uses statistical analysis, Mann-Whiteny U test and Kendall’s *τ* correlation coefficient. The accuracy of the classifier (sensitivity and specificity) is determined by the diagnostic characteristic (ROC). Although the best results are obtained with the Support-Vector Machine (SVM) classifier, the authors strive to find a systematic approach instead of retrospective optimization of results, which will bring better stability in the diagnosis of IHD regardless of the type and distribution of stenosis. The reported sensitivity and specificity for recent studies are 72.1 and 76.3%, respectively.

#### 4.1.2 Myocardial Infarction

Myocardial infarction (MI), also known as a heart attack, is a consequence of coronary artery occlusion and insufficient blood supply to the myocardium and may occur in different parts of the heart. MI triad: ischemia, injury and necrosis or any of the three may appear alone or in combination Dubin (2000). It is reported by Lloyd (2009) that nearly 452,000 Americans die from MI each year and almost every 34 s in the US a heart attack occurs. Coronary artery disease was considered to be the leading cause of death in America in 2004. Accurate detection of myocardial infarction is critical to early medical intervention and improved quality of life. A commonly used method for identifying cardiovascular diseases is time domain ECG, which is now considered as the gold standard. However, projection of cardiac electrical activity in the temporal region will reduce important spatial information about cardiac pathological behaviors. Therefore, new methods using vectorcardiographic recordings that suppress the redundancy of the 12-lead ECG and provide spatial information of cardiac electrical activity for early identification of cardiovascular diseases are being approached.

**FIGURE 4:**
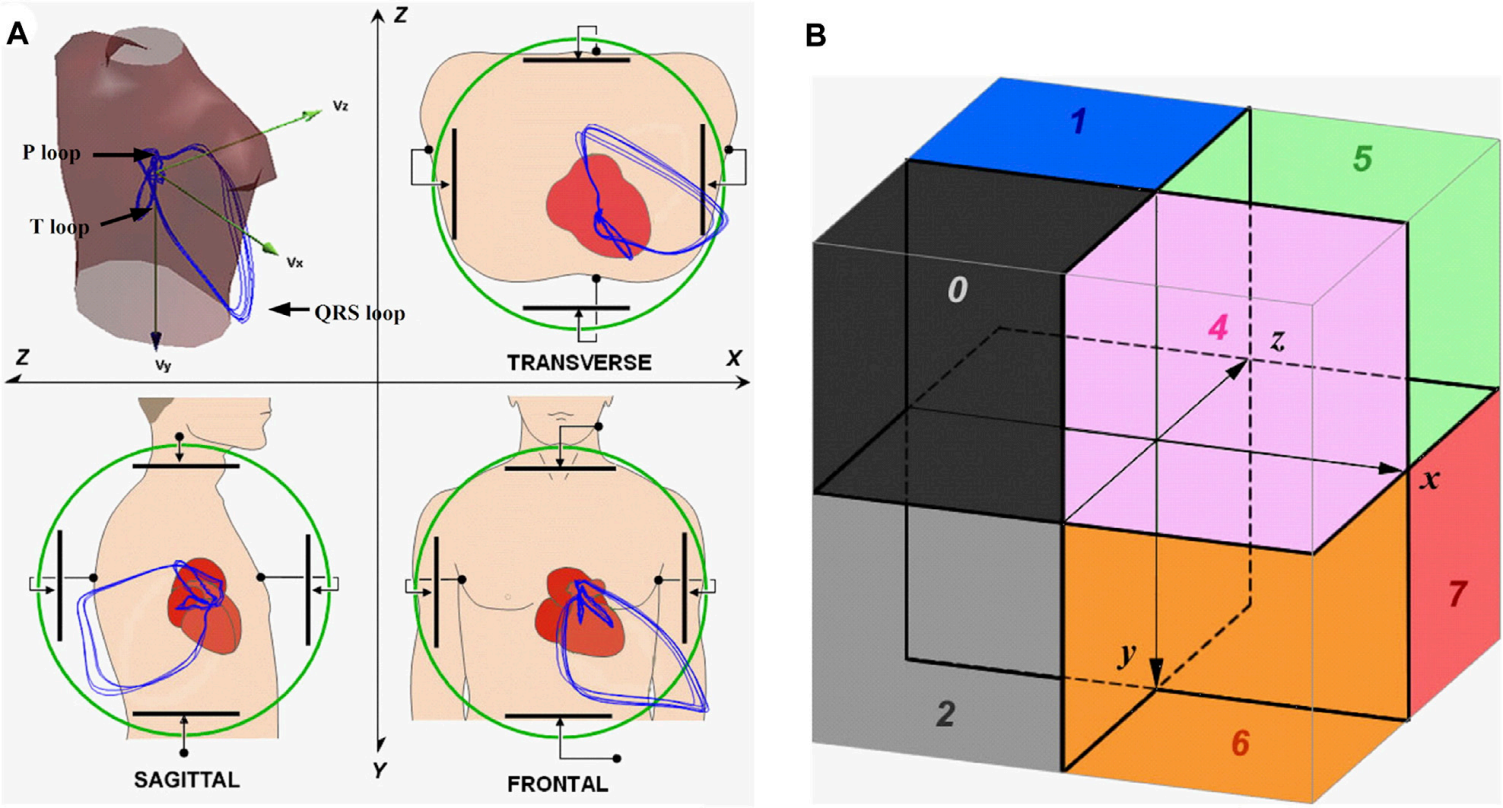
**(A)** Acquiring VCG signals; **(B)** Locations of the eight octants Yang et al. (2012).

VCG loops contain almost periodic activities of P, QRS and T waves in three-dimensional space. This space can be divided into eight octants, defined by Laufberger (1982), which was used by Yang et al. (2012). They presented an approach that uses new spatiotemporal features to identify different types of MI. VCG signals were divided into 8 octants, see Figure 4, from which they extracted a total of 48 VCG features. The analysis was performed for a total of 368 MI records and for 80 HC. They used Classification and Regression Tree (CART) analysis to show that the octant traits of VCG can distinguish MI from HC. The proposed method achieved a sensitivity (accuracy of MI identification) of 97.28% with a specificity (accuracy of HC identification) of 95.00%.

In a previous study Yang (2010) examined the quantification of VCG signals to identify cardiac disorders using the discrete wavelet transform (DWT). A total of three classification models were used to separate healthy and MI records: linear, quadratic, and nearest neighbor (KNN). The linear discriminant analysis (LDA) is based on Eq. 9 and assumes that the linear plane serves as a boundary separating the points HC and MI.

$$y=\alpha \cdot S+\epsilon =\alpha_{0}+\overset{n}{\underset{i=1}{\sum }}\alpha_{i}\cdot S_{i}+\epsilon$$ (9)

where *α*_*i*_ are model coefficients and *S*_*i*_ represents input properties. The residual error *ϵ* of the difference between the actual value and the model output determines whether VCG is HC or MI. Quadratic Discriminant Analysis (QDA), based on Eq. 10, assumes that the hyperplane serves as a boundary separating the HC and MI points in the element space.

$$\begin{array}{cc} & y=\alpha \cdot S+S^{T}\cdot \beta \cdot S=\alpha_{0}+\overset{n}{\underset{i=1}{\sum }}\alpha_{i}\cdot S+\\ & +\overset{n}{\underset{i=1}{\sum }}\overset{n}{\underset{j=1}{\sum }}\beta_{ij}\cdot S_{i}\cdot S_{j}+\epsilon \end{array}$$ (10)

where *α*_*i*_, *β*_*ij*_ are coefficients, *S*_*i*_ represents input properties and *ϵ* are residual errors. In the KNN classification model, the authors proceeded to use the Euclidean metric to measure *proximity*. From all the three analyzed classification models it was LDA that has reached the highest accuracy with sensitivity 96.5% and specificity 75%. The LDA classification technique was also used by Correa et al. (2016) with a total of 95 MI patients and 52 healthy patients. They concluded that several ECG and VCG parameters showed significant differences (p-value < 0.05) between healthy and MI subjects and were able to classify MI and HC subjects with a sensitivity 95.8%, specificity 94.2%, and accuracy 95.2%. Another use of the wavelet transform was used by Sharma and Sunkaria (2018), this time to decompose the segmented multi-lead electrocardiogram (ECG) signal into different sub-bands. They extracted and analyzed a total of 10 VCG features. SVM and a KNN were used to classify MI and HC with an overall accuracy of 81.71%, a sensitivity of 79.01% and a specificity of 79.26%. Extraction and analysis of VCG features together with octant theory was also dealt by Hafshejani et al. (2021). The extracted features were applied to the CART classifier. For the classification of MI and HC records, they achieved an accuracy of 99.4% sensitivity of 100%, and a specificity of 98.7%. Octane-based features were able to distinguish between anterior MI and inferior MI with an accuracy of 98.9%, a sensitivity of 98%, and a specificity of 100%.

Similarly, Kan and Yang (2012) introduced a new warping approach that quantifies the pattern difference in spatiotemporal VCG signals. For a total 309 MI and 79 physiological records the proposed multi-class classification achieves an accuracy > 94.7% for separating MI and HC and an accuracy of 96.5% for anterior-related MIs and inferior-related MIs. In the next study, Yang et al. (2013) presents a model based on Self Organizing Map using VCG parameters. This method has a sensitivity of 94.9% and a specificity of 95.7%. Even greater accuracy in MI detection has been reported by Tripathy and Dandapat (2017). They performed a multilevel analysis of the VCG signal using a complex dual-tree wavelet transform, from which they extracted a total of 15 features. The proposed method for detection MI uses the Vector Machine Relevance classifier (RVM) and the multiscale features of VCG signal, where the accuracy is 99.80%, sensitivity 99.67% and specificity 99.90%. The authors also point out that complex wavelet functions can also be used to detect bundle branch block, cardiomyopathy and ventricular tachyarrhythmia pathologies from the VCG signal. Maximum-likelihood classifier (MLC), general linear model (GLM), the nearest neighbor (KNN) and the Support-Vector Machine (SVM) were used by Huang et al. (2011). They used 6 s of recording for the created classification system. They extracted total 64 VCG features and used 369 MIs and 80 HCs patient records. The proposed classification achieved an accuracy 96.96%, sensitivity 99.89% and specificity 92.51%.

In other publications, the authors dealt with the extraction of VCG MI features affected at various sites in the heart Ge (2008), Kan and Yang (2012). Ge (2008) studied the effectiveness of features extracted from VCG measured by Frank lead system and from classical 12-lead ECG to classify healthy patients (HC), patients with acute infarction (AMI) and subacute infarction (SAMI). The author points out the importance of AMI detection for its great clinical significance, where a small improvement of the algorithm can save lives. The properties of VCG are determined using a M-channel multivariable AR model of order P, based on Eq. 11. The classification is performed by the general linear model (GLM). The average detection accuracy of all diagnoses is 98% for VCG and 73% for ECG.

$$Y\left(k\right)=-\overset{P}{\underset{i=1}{\sum }}A\left(i\right)Y\left(k-i\right)+E\left(k\right)$$ (11)

where *Y(k)* is an M-dimensional column vector of observation over time in current research, *E(k)* is a M-dimensional column vector that represents the multivariable AR modeling errors, *A(i)* for *i = 1, 2, … ,* P are M x M matrices of multivariate AR model coefficients to be estimated. The results show a higher accuracy of pathology detection in VCG analysis than in the case of a standard ECG. Also the higher sensitivity of VCG in detection in patients with inferior infarction is mentioned Hurd et al. (1981), Edenbrand et al. (1990).

By evaluating certain sections of the heart cycle, such as PR Korhonen et al. (2013) or ST Kawahito et al. (2003), Eriksson et al. (2007), El Haddad et al. (2016), Fesmire et al. (2002), Romero et al. (2010), Matveev et al. (2007), Hernandez et al. (2018), it is possible to develop identification methods for further detection of heart disease. Study dealing with changes in the PR segment of the heart cycle by Jim et al. (2006) inspired Korhonen et al. (2013) to evaluate dynamic PR segment changes for 37 patients with AMI. They analyzed PR interval, PR level, Change in PR level, PR area and Change in PR area. The findings support the concept that atrial ischemia causes similar changes in the PR segment as ventricular ischemia in the ST segment. Similarly, Eriksson et al. (2007) studied the prognostic value of various reperfusion criteria for short-term continuous vectorcardiography in 400 patients with ST-segment elevation of myocardial infarction. They analyzed ST vector size (ST-VM) at the beginning of registration and at the beginning of thrombolysis, ST-VM 60 and 90 min after the start of thrombolysis, and maximal ST-VM. They show that VCG may be useful in selection of patients for coronary angiography followed by revascularization. El Haddad et al. (2016) developed and validated a novel method for detection transmural ischemia based on a new 3-lead configuration. They found that the proposed 3-lead configuration is highly accurate for the early detection of acute occlusion-related ischemia and exceeds conventional 12-lead ECG criteria. This method provides a platform for self-detection of CAO using handheld devices or smartphones.

In recent years, there has been an increasing move to process VCG features to improve pathology detection. Therefore, Hernandez et al. (2018) analyzed 322 VCG features to improve the detection of AMI from the ST and T wave section which they reduced to five major features by using stepwise forward selection as a method for variable selection. From their results, they found that VCG features are able to diagnose AMI slightly better than ST elevation and T-wave maximum features together. Goernig et al. (2015) dealt with T vector and T-loop parameters compared to a conventional 12-lead ECG. They used 114 patient records and analyzed T vector elevation, T vector azimuth, QRS-T angle, *T*_*area*_, *T*_*eigenv*_ (expresses the form and the symmetry of the T loop) and *T*_*avplan*_ (expresses the bulginess of the loop). MI detection was successfully identified in 75.2% of ECG records and 83.2% of T vector and loop parameters from VCG.

Romero et al. (2010) assess depolarization changes during AMI using analysis of the QRS loop represented by principal component analysis (PCA). In addition to the classical method based on the analysis of the ST-T section of ECG leads, it thus provides a new vector approach to the detection of AMI. The method includes signal preprocessing, which includes QRS detection, detection of normal cardiac cycles, isolation of the isoelectric line by cubic spline interpolation (CSI), delineation using wavelet (WT) techniques, and normalization. The result is an improvement in the sensitivity of the ST-segment inclination parameters by 103%, respectively 46% for the VCG method compared to the ECG for AMI detection.

Aranda et al. (2015) made a comparison between transformed VCG records using IDT and originally measured VCG to identify which record achieves better MI detection results. In conclusion, the authors present two results, namely that the inverse Dower transform achieves the same MI detection success rate as Frank lead system and the second result, generally known, that electrode positions have a great influence on the accuracy of identifying patients with MI.

Chen and Yang (2013) developed a multi-step repeat approach to VCG analysis for MI detection. Analysis of multiple recurrences of VCG signals resulted in an improved classification model having an average sensitivity of 96.8% and a specificity of 92.8%. The time of recording also plays an important role in the success of arrhythmia detection, therefore Kulbertus and de Leval-Rutten (1974) described the vectorcardiographic features observed during true apical ventricular pacing in myocardial infarction patients. He states that deformities may gradually decrease in later stages of the disease. New method for processing angular acceleration during ventricular repolarization from Frank lead system was proposed by Cruces and Arini (2016). By combining the linear velocity, obtained by differentiation, and the spatial velocity shown during ventricular depolarization, they created a new VCG feature (the so-called Heart Vector Rate Index: ICVV) with high sensitivity (92%) and specificity (97%) values.

The processing of vectorcardiographic features has become a reliable and recently expanded tool for the analysis of the VCG curve. The creation of new features or improvements in analysis techniques can contribute to better processing of VCG data and achieve early detection of heart disease. The new VCG feature was also proposed by Sadhukhan et al. (2017), which consists of a combination of the volume ratio of the 3-D QRS and the ST-T loop. From a statistical analysis, they found that this new feature provide high sensitivity (98.8%) between the MI and HC groups. Keshtkar et al. (2013) analyzed VCG features for 50 MI and 50 HC records and their proposed method based on probabilistic neural networks achieves 93.0% sensitivity at 86.0% specificity with 89.5% accuracy.

There are also papers that point to a significant impact of a combination of QRS complex and ST segment analysis Treskes et al. (2015), van Hellemond et al. (2013). Treskes et al. (2015) analyzed ST vector at J-point (J-point is automatically localized when the heart vector between QRS and T wave reaches its minimum value) and ventricular gradient vector (VG) from modified VCG by Kors regression method. According to their findings, the authors report that ST and VG analysis for the detection of acute myocardial ischemia is feasible and has either the same or even better performance than the conventional method. Also, van Hellemond et al. (2013) based on their QRS and ST parameter evaluation study, the myocardial risk area (MaR) estimation was more accurate that both ST and QRS abnormalities were taken into account than using individual components alone. The morphology of QRS-T for MI identification was also addressed by Hasan et al. (2012b), where they investigated beat-to-beat variations in their work and they find beat-to-beat analysis useful for characterizing abnormalities in MI patients. They further expanded their work in extracting other features and further supported their previous claims Hasan et al. (2012b). Their proposed algorithm has been further improved in robustness, which now achieves better results even on noisy data Karisik et al. (2019). Based on these results, it can be argued that beat-to-beat VCG analysis can be considered useful for distinguishing MI.

### 4.2 Myocardial Scar

Fatal arrhythmias (ventricular tachycardia, fibrillation, etc.), most commonly caused by conduction defect from injured cardiac tissue known as scar, are a major cause of sudden cardiac death. Myocardial scar is the result of myocardial infarction (MI), which is caused by insufficient blood supply to the heart. This results in the death of part of the heart cells (scar tissue) and affects the contractile properties of the myocardium. Methods for accurate localization and scar size determination are different imaging techniques such as magnetic resonance imaging (MRI). However, this presents a number of disadvantages such as limited availability, high costs and mobility. Therefore, new methods for early identification of scar tissue in patients suffering from MI are being approached.

In 1971, Selvester et al. (1971) described detailed ECG and VCG criteria developed from simulations for localization and quantification of MI size. From these simulations, it was found that in a lead at right angles to the epicardium of the respective area, the degree of QRS change was proportional to the size of the infarct. Wickline and McNamara (1978) confirmed this finding based on autopsy results performed on baboons and Selvester et al. (1979, 1982) correlated the ECG/VCG QRS MI size scores with biplane ventriculograms that of the LV. The VCG score was calculated based on the deformations in the amplitude and length of the QRS applied to the nomogram developed in the set-learning test mode to predict the size of the MI. The 12-lead ECG MI size score involved 57 criteria (Q- and R-wave duration, R- and S-wave amplitudes, R/Q ratios, R/S ratios, and QRS slurs or notches) in 10 of the 12 standard leads except aVR and III. The maximum number of points was 32, and each point corresponded to 3% of the LV mass (representing 96% of the LV) Strauss and Selvester (2009).

VCG features are typically used for automatic classification using machine learning algorithms. For example, Panagiotou et al. (2013) used 27 spatial features to detect the presence or absence of a scar in myocardial tissue. For processing they used modified VCG by using IDT. For a total of 46 records, they developed a classification scheme based on SVM for scar detection. The proposed method achieves an accuracy of 82.36% (sensitivity 84.31% and specificity 77.36%). Another method for scar detection using a complex model based on the SVM was presented by Dima et al. (2013). A group of extracted features from ECG and derived VCG was used to formulate a classification model of the machine model of supportive learning. From a total of 260 used patient records, this model achieved an accuracy of 82.07% (sensitivity 76% and specificity 87.5%).

Nguyên et al. (2018) investigated the relationship between VCG parameters and myocardial scar (focal and diffuse) on CMR in patients with heart failure with ventricular dysfunction and whether the combination of VCG with scar parameters obtained by CMR improves the prediction of CRT response. They extracted *QRS*_*area*_, *T*_*area*_ and *QRST*_*area*_ from the derived leads using the Kors regression method. Based on their findings, it follows that focal scar CMR parameters and QRSarea are independent predictors for CRT response and are inversely associated with each other. The highest percentage of CRT response was observed in patients with low focal scar CMR values and high QRSarea, indicating that combined CMR-VCG parameters may improve prediction to CRT response. The issue dealing with *QRS*_*area*_ and CMR was also dealt by Okafor et al. (2020). The purpose of their study was to determine in patients with ideally deployed quadripolar left ventricular lead whether a reduction *QRS*_*area*_ leads to an acute hemodynamic response (AHR) and whether the scar affects this interaction. In their study, they found that lowering the *QRS*_*area*_ improved AHR to CRT, myocardial scar adversely affects *QRS*_*area*_ and AHR.

Bizarro et al. (1978) performed a multivariate discriminant procedure for VCG identification of ischemic myocardial scars from 1162 HC records and 90 VCGs obtained from patients proved at autopsy to have ischemic myocardial scars. They used a total of 16 VCG features for discriminant analysis, which achieved sensitivity 90% and specificity 97.2%. The vectorcardiogram was also used as a non-invasive remodeling method to detect the presence, location, and dimensions of cardiac scars. Using a clinically derived computational model of the entire torso, Gemmell et al. (2020) performed both slow and fast pacing simulations for various myocardial scar patterns. Their results show that differences in the dipole angle at the end of the QRS complex and differences in QRS area can be used to predict scar properties and were also be able to predict the location of the scar with high accuracy.

Approximately 80% of the causes of SCD are associated with coronary heart disease Myerburg and Junttila (2012). The possibility of finding a suitable marker for SCD identification was analyzed by Waks et al. (2015). They analyzed beat-to-beat spatiotemporal variability in the T vector as the mean angle between consecutive T-wave vectors. They applied IDT transformation to derive VCG leads to the measured ECG recordings. Based on their findings in a large study, the increased risk of SCD is associated with higher variability in temporal and spatial variability between beats in the spatial T vector. In a previous study Tereshchenko et al. (2010) also looked at beat-to-beat analysis of VCG records now to predict ventricular arrhythmia (AV) as a possible predisposition to SCD. From their analysis, the authors state that T peaks are predictors associated with an increased risk of ventricular arrhythmias.

### 4.3 Left Bundle Branch Block

Left Bundle Branch Block (LBBB) is a disorder of cardiac conduction in the myocardium. Rather than heart disease, it is an abnormality of intraventricular conduction where the left ventricle is abnormally activated from the right bundle branch which starts branching in the apical region. The entire left ventricle is depolarized from the right bundle branching in the apical region, thereby expanding and morphologically changing the QRS complex. The main feature of LBBB is the mismatch between the main axes of the QRS complex and the T-wave, which is consistent with very wide QRS-T angles. Frank lead system can be considered the gold standard for precisely defining the mean spatial and vertex angles of QRS-T Schreurs et al. (2010). In patients with LBBB and heart failure, the goal is to achieve a more synchronous model of electrical ventricular activation and contraction by left ventricular-based stimulation (LV), thereby improving LV systolic function. Therefore, van Deursen et al. (2015a) and van Deursen et al. (2015b) utilized vectorcardiography knowledge to more accurately select patients for cardiac resynchronization therapy (CRT). They evaluated the QRS region from vectorcardiography against the commonly used QRS duration and left bundle branching morphology. Their findings suggest that the QRS area is a stronger predictor of CRT compared to the QRS time or conventionally defined LBBB morphology. A more precise selection of candidates for CRT was also dealt by Rad et al. (2016), where the main parameter of the study was also the QRS area. They used a total of 51 VCGs derived by Kors regression method. They compared the QRS area with QRS length and, based on their analysis, conclude that the QRS area is a non-invasive alternative to intracardiac measurement of electrical activation that identifies delayed left ventricular lateral wall activation better than QRS duration and LBBB morphology.

Shvilkin et al. (2010) in their study they distinguished the criteria to differentiate new and old LBBB. IDT were applied to the measured ECG recordings to obtain derived VCG leads. They analyzed QRS and T-wave amplitudes, directions, and durations and found that the QRS/T vector magnitude ratio and the deepest S to largest T wave ratio allow accurate discrimination between the new and old LBBBs with 100% sensitivity and 96–68% specificity. This finding may help management of patients with chest pain and LBBB. In another study, Villongco et al. (2014) described a method for simulating LBBB branches and RV-stimulated ventricular activation profiles in three dimensions from non-invasive routine clinical measurements. They used IDT and Kors regression transformation to derive VCG. They developed an activation pattern predicted by optimal parameters that correlates with invasive endocardial activation time measurements. The aim of this approach is to improve non-invasive electrocardiographic imaging techniques.

### 4.4 Hypertrophic Cardiomyopathy

Hypertrophic cardiomyopathy (HCM) is a cardiac disorder with heterogeneous expression, specific pathophysiology and a diverse clinical course. Several disease-causing mutations have been reported in genes encoding sarcomere proteins. Due to its characteristic clinical, morphological and genetic diversity, hypertrophic cardiomyopathy has maintained the curiosity of physicians and scientists for almost 40 years Maron (1997). Whereas HCM is the most common cardiomyopathy, left ventricular hypertrophy is, however, more commonly due to hypertension and VCG might be useful here mainly due to QRS and T mismatch and more circular T-loop.

One of the first studies at HCM was conducted by Chen and Kawai (1978), where they examined a total of 45 patient VCG records and their QRS and T loops. In its findings, it draws attention to the changes in loops between HCM and HC.

Current ECG criteria have low accuracy in the diagnosis of left ventricular hypertrophy (LVH). However, the magnitude of QRS vectors and the direction of T loops can be considered useful parameters for the diagnosis of LVH from VCG MURATA et al. (1964). Man et al. (2012) trying to improve the diagnostic accuracy of LVH by combining demographic, anthropomorphic ECG and VCG variables. They used 196 VCG records derived by Kors regression method. They divided the study group into four subgroups and compared patient characteristics in each subgroup with an unpaired *t* test or a *χ* ^2^ test. They created a discriminatory model based on Eq. 12, where D greater or equal than 0 predicts normal echocardiogram and D less than 0 predicts LVH.

D=5,130⋅BSA−0,014⋅SA−8,74 (12)

where the *BSA* corresponds to the body surface area and *SA* spatial QRS-T angle. The diagnostic accuracy (79%) was better than the diagnostic accuracy of the conventional LVH ECG criteria (57%). The VCG was also analyzed by Kataoka and Tomoike (2021) to increase the accuracy of LVH diagnosis. Their goal was to quantify the three-dimensional characteristics of the P, QRS, and T loops from which they extracted features using the minimum volume ellipsoid enclosure (MVEE). They used random forests as a decision classifier for the extracted features, where they achieved an accuracy of 0.904 (95% confidence interval: 0.861–0.947).

HCM is a genetic heart disease with unexplained left ventricular hypertrophy and is one of the most common causes of sudden cardiac death in young people. The issue of HCM was also studied by Cortez et al. (2017b) and used a total 967 VCG records derived by Kors regression method. They analyzed spatial mean and peaks QRS-T angles, spatial ventricular gradient (SVG), spatial QRS, QT, and *T*_*peak*_-*T*_*end*_ (*T*_*p*_ *T*_*e*_) intervals. They evaluated the differences between patients with HCM with a positive genotype and with a negative genotype. They found that the QRS-T parameter from derived VCG can distinguish patients with HCM from HC.

The HCM study in children was also studied by Jimenez et al. (2021), where they analyzed the T loop from the derived VCG. Based on T-wave vector (VM) analysis, it was found that lower VM was associated with a higher risk of developing a sentinel event in HCM, presumably as an indicator of abnormal repolarization.

### 4.5 Long QT Syndrome

Long Q-T syndrome (LQTS) is an inherited and life-threatening condition whose initial symptoms develop in childhood or adolescence. Ion channel dysfunction in myocytes causes delayed repolarization. The characteristic prolonged Q-T interval is associated with *torsade de pointes* and sudden cardiac death Diamant et al. (2013), Goldenberg and Moss (2008).

Important finding was presented by Diamant et al. (2013, 2010) in the diagnosis of long QT syndrome from vectorcardiography. Diamant et al. (2013) determined whether the VCG record was better than a 12-lead electrocardiogram in providing a correct diagnosis of long QT syndrome (LQTS) in children. For their study, they used a set of 70 child records, where 35 records were diagnosed with long QT syndrome. Of 35 children with genetically confirmed LQTS, 30 (86%) were diagnosed correctly with *QT*_*VCG*_ and 24 (69%) with *QT*_*ECG*_. The specificity was 80% for *QT*_*VCG*_ and 77% for *QT*_*ECG*_. While Diamant et al. (2010) presented two methods of QT analysis, manual evaluation from ECG using four experienced observers and automatic computer measurement with evaluation from VCG. They confirm that automatic VCG detection achieves high precision and reliability in LQTS detection. From these findings, they indicate the importance of vectorcardiography in the detection of LQTS.

Cortez et al. (2017a) investigated by retrospective analysis patients with hidden LQTS (ecLQTS). They used a dataset out of a total of 610 records with LQTS (169 records with ecLQTS) compared to 519 records of healthy individuals. They found that ecLQTS can be distinguished from healthy individuals by QT peak analysis.

### 4.6 Atrial Fibrillation

Atrial fibrillation (AF) is one of the most common heart diseases occurring in 1–2% of the population. Although AF itself is not a life-threatening condition, it increases the risk of stroke fivefold, and every fifth stroke is attributed to this arrhythmia. Ischemic strokes associated with AF are often fatal, and patients who survive are more affected by stroke and are more likely to relapse than patients with other causes of stroke Camm et al. (2010).

New methods for correct AF detection were sought. Bartall et al. (1978) compared the accuracy of AF detection from a standard 12-lead ECG and a VCG. They concluded that VCG measured by Frank lead system achieves a higher sensitivity for the detection of echocardiographically detectable left atrial enlargement and a higher success rate of AF detection. Filos et al. (2017) dealt with AF which can last up to 48 h. They investigated patterns of P-wave morphology that may occur in patients with Paroxysmal AF (PAF). They used 29 PAF and 34 physiological VCG records using a method based on the identification of dominant and secondary P-wave morphology by a combination of adaptive clustering of morphologies (KNN) and the beat-to-beat correlation technique. From their analysis, seven features were highlighted, which the SMV classifier determined the detection accuracy at 93.75%. The issue of PAF was also examined by Filipova et al. (2017) and Yamashita et al. (2011). Filipova et al. (2017) studied total 276 patients with a diagnosis of hypertension (HT) at risk for paroxysmal atrial fibrillation. It is possible, that especially P wave and P loop values can reflect the abnormal status in atrial myocardium prior the PAF onset. Their analysis shows that HT patients with verified PAF have more abnormal P and QRS wave and loop parameters than HT patients without a history of PAF.

The problem of early detection of AF was also addressed by Kawano et al. (1988). In their study, they found that P waves from patients with transient AF without apparent anatomical changes in the atria showed abnormal findings in the VCG. These abnormal phenomena include: 1) Prolonged duration and increased amplitude of the P wave in recordings from Frank lead system 2) Abnormal bites or notches in the P loops in the VCG 3) Increased values of the P waves in the spatial velocity ECG and spatial magnitude ECG 4) Prolongation of both the anterior and the left maximum in the body surface P maps. For better analysis during AF, van Oosterom et al. (2007) designed a vectorcardiographic lead system based on the Gabor-Nelson equation. Six electrode configurations and their dedicated transfer matrices were tested using 10 different episodes of simulated AF and 25 different chest geometries. Their results indicate that the alternative electrode configuration should include at least 1 electrode on the back of the body to observe the dipolar activity of the atria during AF.

Transformation methods have also found application in the analysis of spatial characteristics of AF from derived VCG using IDT Carlson et al. (2005); Ng et al. (2004); Guillem et al. (2009). Ng et al. (2004) analyzed the synthesized VCGs of each f-wave cycle of each ECG and its plane of best fit, described by azimuth and elevation angles relative to the frontal plane. From their findings, fifteen of the 22 patient records analyzed had at least 30% of the planes in one 30-degree region of azimuth angles. While Guillem et al. (2009) compared the loop orientation of the derived and directly measured VCG. They state from their results that IDT should not be used for the analysis of fibrillatory wave loops in AF, due to the fact that the spatial parameters can differ significantly from the directly measured VCG. However, it should be borne in mind that the transformation methods also work with the presence of the P wave, and in the case of AF, the transformation could introduce a significant error into the derived signal.

However, AF can also be reliably detected on a standard ECG, but there may be some problems when using one or a few leads. In this respect, VCG could provide support in applications where fewer leads are used in a 12-lead ECG, such as patient monitors or smartphones or watches. The use of a lower number of leads can also be used in VCG, where it is necessary to focus on the direction of propagation of the potential P wave vector.

## 5 Current Problems

The identification of heart disease from diagnostic methods is a frequently discussed topic in recent years. For decades, 12-lead ECG has been considered as the gold standard for electrical heart activity diagnostics. However, there are cases where a standard ECG does not achieve the necessary success rate of detecting various ischemia using automated detection. Therefore, VCG is approached as a method that can provide additional information Edenbrand et al. (1990), Stovicek et al. (1993), Warner et al. (1982), Filos et al. (2017), Iwaniec et al. (2018), Okafor et al. (2020), Jimenez et al. (2021). Standardized VCG leads using Frank lead system are rarely used nowadays, and for this reason trends are shifting to a derivation solution. Comparative studies and individual work have shown that regression transformations are more accurate than model-based transformations Kors et al. (1990), Levkov (1987), Rubel et al. (1991), Jaros et al. (2019). Many different regression transformation matrices can be obtained, all of which yield similar results. Quasi-orthogonal leads are not truly orthogonal and the interpretation of VCG is only approximate. Although there is some correlation with VCG leads, the spatial information that can be obtained from the ECG is reduced. In some studies, it has been shown that these Quasi-orthogonal derivation methods achieve poor results Kors et al. (1990), Levkov (1987), Rubel et al. (1991) and for these reasons they should not be used for further processing.

For the processing of pathological data, there may be a problem in selecting the most suitable transformation method. The reliability of the methods is also conditioned by different and inconsistent evaluation parameters. Linear transformation methods have proven to be a useful and frequently used tool for converting 12-lead ECGs to VCGs. The most commonly used transformation methods include the IDT, the Kors regression, the PLSV and QLSV methods, and in some cases also the Quasi-orthogonal approach. IDT is the most commonly used transformation method for further processing. This transformation method is mainly focused on the QRS complex, but also achieve reliable accuracy in other parts of the ECG signal. Methods that have been derived to improve accuracy only for some parts of the cardiac cycle (QLSV, PLSV) achieve the same accuracy as the regression approach. However, the accuracy of transformation methods also needs to be evaluated with a cardiologist. The problem with transformation methods and their transformation accuracy, in which the preservation of diagnostic information is addressed, would be solved by more frequent use of Frank lead system in clinical practice.

As additional information for the detection of heart disease are used VCG features. Studies have shown that VCG features extracted from pathological records such as myocardial infarction, myocardial ischemia, myocardial scar or atrial fibrillation can reliably distinguish test groups from healthy controls. Current studies have confirmed that the VCG features analysis especially of the QRS complex in particular provides reliable detection results Correa et al. (2010); Yang et al. (2012); Tripathy and Dandapat (2017); Kan and Yang (2012). An overview of selected important comparative studies dealing with VCG features are summarized in Table 9. The relevant table describes the scientific work relevant in particular to this review. Central to the table, is the column *VCG features*, which shows the total number of VCG features for individual works and their most important features. For publications with a large number of features, only the number is given.

**TABLE 9:** An overview of important comparative studies.

Author	Year	Purpose	VCG features	Data collection	Transformation method
Kawahito et al. (2003)	2003	QRS, ST-T parameters study	2 features QRS vector difference, ST vector magnitude	VCG measured by authors	none
Huang et al. (2011)	2011	MI detection	64 features, QRS, T vector magnitudes R-T peak angle	PhysioNet PTB	none
Yang et al. (2012)	2012	MI detection	48 features Q, R, T—vector magnitude, R, T—vector angle, Angle between R and T-vector	PhysioNet PTB	none
Correa et al. (2012)	2012	QRS, ST-T parameters study	8 features QRS—Volume, Planar Area, Ratio between Area and Perimeter, Perimeter, ST Vector Magnitude, ST segment Level, T-wave amplitude	PhysioNet PTB	Kors regress
Correa et al. (2013)	2013	Cardiac ischemia detection	8 features	PhysioNet PTB	Kors regress
Panagiotou et al. (2013)	2013	Myocardial scar detection	27 features R-width, T-width magnitude, R-peak, T-peak	PhysioNet PTB, Cardiology Department (UHS-NHS)	Dower’s inverse
Dima et al. (2013)	2013	Myocardial scar detection	25 features	PhysioNet PTB, Cardiology Department (UHS-NHS)	Dower’s inverse
Treskes et al. (2015)	2015	Myocardial ischemia detection	2 features ST vector, Ventricular gradient vector	ECG measured by authors	Kors regress
Aranda et al. (2015)	2015	MI detection	98 features	PhysioNet PTB	Dower’s inverse
Correa et al. (2016)	2016	MI detection	9 features QRS—Volume, Planar Area, Perimeter, Vector difference in ST segment and T-wave, ST-T Vector Magnitude Difference	PhysioNet PTB	none
Sedaghat et al. (2016)	2016	QRS parameters Study	5 features QRS loop roundness, planarity, thickness, rotational angle, dihedral angle	ECG and VCG measured by authors	Dower’s inverse
Sun et al. (2017)	2017	Myocardial ischemia screening	17 features	PhysioNet PTB, STAFF III	Dower’s inverse
Tripathy and Dandapat (2017)	2017	MI detection	15 features	PhysioNet PTB	none
Sharma and Sunkaria (2018)	2018	MI detection	10 features	PhysioNet PTB	none
Gemmell et al. (2020)	2020	Myocardial scar detection	6 features	Derived whole-torso computational model with simulations	none
Hafshejani et al. (2021)	2021	MI detection	48 features	PhysioNet PTB	none

Paramount is to evaluate the diagnostic significance of individual features, which may be different for different pathologies. Most studies only deal with the individualization of VCG features to a particular dataset. Thus, their results do not have to correspond to another database. It would be useful to compare the effectiveness of different features between the databases because each dataset is obtained by different measurement parameters. This fact is also correlated to the small number of available VCG datasets, where only a few authors use their own measured records for their study. The lack of records could be reduced by providing records by the authors or creating new datasets using modern devices. There are VCG features that are used in most publications and achieve high detection accuracy in terms of statistical evaluation. However, these features are not standardized for certain arrhythmias despite their possible diagnostic benefit, caused mainly by not consulting the results with cardiologists. The derivation and extraction of additional VCG features could further contribute to obtaining additional information necessary for the early diagnosis of heart disease.

## 6 Conclusion

Vectorcardiography, which in recent years is more often analyzed mainly in the research field, and which represents a different approach in the representation of electrical activity of the heart, can help us to obtain information that can help in the early diagnosis of heart disease. The main problem is that this method is not measured in clinical practice and the authors approach this method using transformation methods. The most important condition for the transformations is that diagnostic information must not be lost. Only an experienced cardiologist can evaluate the effect of transformation. If we only need a certain part of the heart revolution for processing, it is more appropriate to use specialized methods, which are focused on that part. The most accurate current methods are the IDT and the Kors regression transform but they lag slightly behind the accuracy of the P and T waves. This gap in the imperfection of linear transformation methods needs to be further addressed, due to the consequent better accuracy of transformation and preservation of diagnostic information. The new transformation method could refine VCG findings and provide physicians additional information for the early treatment of heart disease. Complications of applying transformation methods could be reduced by more frequent use of Frank lead system in clinical practice.

In most cases processing of VCG is performed on already measured datasets and is performed so-called offline. Future intentions may lead to the creation of devices that will process data using already proven techniques in real time or on newly created data or datasets. To detect different pathologies, the authors use different VCG features applied to derived or directly measured VCGs. Certain features are often pointed out that achieve reliable pathological detection. The authors analyze various parts of the cardiac revolution, such as the QRS complex, where they achieve relatively high detection accuracy (sensitivity and specificity is often > 90%) or the S-T region with sensitivity and specificity around 80%. However, different processing methods for each access are often used. To complete the knowledge, it would be appropriate to focus on a certain type of processing. Few authors also consult their results with cardiologists to confirm their importance. They merely point out differences in morphological properties to assess the new method. The assessment of certain features by cardiologists could help standardize the features for certain pathologies. Combinations of VCG features for different loops of cardiac cycle achieve promising results but this approach is rarely used. By combining the QRS complex and the ST segment features, greater differences can be achieved between groups HC and the pathology. It would be advisable to consider using multiple VCG parts (QRS loop, ST segment, T wave) for analysis rather than just one.

Combining the above suggestions and discussing the results with cardiologists could help with visualization and gain new insights into the electrical activity of the heart. It would also be beneficial to increase the frequency of use of Frank lead system in clinical practice because as has been shown by more accessible and improving computer technology, the VCG method is suitable for the analysis of various heart diseases.
